# *CD40LG* and *GZMB* were correlated with adipose tissue macrophage infiltration and involved in obstructive sleep apnea related metabolic dysregulation: Evidence from bioinformatics analysis

**DOI:** 10.3389/fgene.2023.1128139

**Published:** 2023-02-27

**Authors:** Xiaoping Ming, Weisong Cai, Zhen Li, Xiuping Yang, Minlan Yang, Dingyu Pan, Xiong Chen

**Affiliations:** ^1^ Department of Otorhinolaryngology, Head, and Neck Surgery, Zhongnan Hospital of Wuhan University, Wuhan, China; ^2^ Sleep Medicine Center, Zhongnan Hospital of Wuhan University, Wuhan, China; ^3^ Department of Hepatobiliary and Pancreatic Surgery, Zhongnan Hospital of Wuhan University, Wuhan, China; ^4^ Bariatric and Metabolic Disease Surgery Center, Zhongnan Hospital of Wuhan University, Wuhan, China

**Keywords:** obstructive sleep apena, adipose tissue–obesity, macrophage infiltration and polarization, *CD40L* CD-40-ligand, *GZMB* Granzyme B, review, fat, metabolic dysfunction

## Abstract

Both obesity and obstructive sleep apnea (OSA) can lead to metabolic dysregulation and systemic inflammation. Similar to obesity, increasing evidence has revealed that immune infiltration in the visceral adipose tissue (VAT) is associated with obstructive sleep apnea-related morbidity. However, the pathological changes and potential molecular mechanisms in visceral adipose tissue of obstructive sleep apnea patients need to be further studied. Herein, by bioinformatics analysis and clinical validation methods, including the immune-related differentially expressed genes (IRDEGs) analysis, protein-protein interaction network (PPI), functional enrichment analysis, a devolution algorithm (CIBERSORT), spearman’s correlation analysis, polymerase chain reaction (PCR), Enzyme-linked immunosorbent assay (ELISA) and immunohistochemistry (IHC), we identified and validated 10 hub IRDEGs, the relative mRNA expression of four hub genes (*CRP, CD40LG, CCL20, and GZMB*), and the protein expression level of two hub genes (*CD40LG* and *GZMB*) were consistent with the bioinformatics analysis results. Immune infiltration results further revealed that obstructive sleep apnea patients contained a higher proportion of pro-inflammatory M1 macrophages and a lower proportion of M2 macrophages. Spearman’s correlation analysis showed that *CD40LG* was positively correlated with M1 macrophages and *GZMB* was negatively correlated with M2 macrophages. *CD40LG* and *GZMB* might play a vital role in the visceral adipose tissue homeostasis of obstructive sleep apnea patients. Their interaction with macrophages and involved pathways not only provides new insights for understanding molecular mechanisms but also be of great significance in discovering novel small molecules or other promising candidates as immunotherapies of OSA-associated metabolic complications.

## 1 Introduction

Obstructive sleep apnea (OSA), accounting for one-seventh of the world’s adult population, is a condition of apnea and hypopnea caused by the collapse of the upper airway during sleep, accompanied by snoring, disturbance of sleep structure, frequent oxygen desaturation, and daytime sleepiness (Benjafield et al., 2019). OSA is particularly common in obese people, and its incidence is increasing in parallel with the obesity pandemic (Bonsignore 2022). Long-term suffering from the disease can lead to systemic inflammation and metabolic dysregulation, such as cardiovascular disease, type 2 diabetes, non-alcoholic fatty liver disease, and hyperlipidemia (Punjabi 2008; Priou et al., 2012; Tan et al., 2014; Parikh et al., 2019). The interaction between these conditions has a momentous effect on patient care and mortality (Lyons et al., 2020). In addition, Continuous positive airway pressure is the primary treatment for adult OSA, but its efficacy in improving cardiovascular and metabolic outcomes is lacking (Light et al., 2018). Taking all those aspects together, it is of great significance in identifying the molecular mechanisms involved in OSA development and in subsequently discovering therapies for OSA-associated metabolic complications.

Although OSA patients have a 2-3 fold increased risk of developing multiple end-organ morbidities, none of them emerge any specific end-organ dysfunction (Kheirandish-Gozal & Gozal 2019). An intense investigation of systemic inflammation as a contributing factor to OSA-related morbidity has been conducted because of the heterogeneity of the clinical phenotype (Drager et al., 2010; Bhattacharjee et al., 2011; Gozal et al., 2012; Kheirandish-Gozal & Gozal 2013; Drager et al., 2015). Chronic intermittent hypoxia, one hallmark features of OSA, can preferentially activate NF-κB mediated proinflammatory signaling pathway, leading to a systemic inflammatory state in OSA patients, but little is known about the tissues that produce pro-inflammatory mediators in reaction to OSA (Murphy et al., 2017). As both obesity and OSA can lead to similar metabolic complications, it is reasonable to speculate that adipose tissue may be one of the target tissues in response to proinflammatory mediators. Adipose tissue is not only an organ of storing energy but also a highly active endocrine organ regulating metabolism (Ryan et al., 2019). Evidence from rodent experiments showed that intermittent hypoxia in OSA induced insulin resistance and atherogenesis through fat inflammation (Poulain et al., 2014; Murphy et al., 2017). Similar to obesity, adipose tissue macrophages play a vital role in intermittent hypoxia-induced fat inflammation (Poulain et al., 2014; Murphy et al., 2017). However, the largest and by far best-studied parts of fat are located in the gonadal region of rodents, there is no similarity to these pads in humans (Ryan et al., 2019). Hence, we cannot be easily translated the findings obtained from rodent studies into human conditions.

To date, most of the research on human adipose tissue has been focused on the field of obesity, while research on the OSA subject is relatively rare due to the lack of attention to OSA condition, multi-disciplinary limitations, and ethical factors in a routine sampling of adipose tissue in humans. Aron-Wisnewsky J et al. found no relationship between OSA and *HAM56*-labeled total adipose tissue macrophage infiltration by sampling omental adipose tissue during bariatric surgery (Aron-Wisnewsky et al., 2012), but they did not analyze the detailed phenotyping of the macrophages. Grab et al. showed that OSA can alter fat gene expression particularly in metabolic dysregulation by analyzing the transcriptomic profile of subcutaneous and visceral fat in humans (Gharib et al., 2013; Gharib et al., 2020). They also found that “Immunity and Inflammation” was one of the most upregulated modules in OSA, but detailed information about inflammatory immune-related gene expression profiles and their interaction with immune cell infiltration needs to be further studied.

In the current study, we assessed the microarray dataset GSE38792 that contains visceral adipose tissue (VAT) of OSA patients from Gene Expression Omnibus (GEO) and carried out an integrated bioinformatics analysis. The components of immune infiltration in VAT of OSA patients were also analyzed using the CIBERSORT algorithm method (Newman et al., 2015; Kawada et al., 2021). More importantly, VAT samples from obese individuals with complete overnight polysomnography (PSG) examination (the gold standard for the diagnosis of OSA) were obtained for external validation through multidisciplinary approach. To our knowledge, this is the first study that using clinical samples to validate the findings of dataset analysis, which provides direct evidence for adipose tissue as a proinflammatory target organ for OSA-associated metabolic complications. The aim of this study was to identify and validate the immune-related differentially expressed genes (IRDEGs) and the characteristics of immune infiltration in VAT of OSA patients and to provide new knowledge for understanding molecular mechanisms of OSA-induced metabolic complications.

## 2 Materials and methods

### 2.1 Ethics statement

The study protocol was ethically authorized by the Ethics Committees of Zhongnan Hospital of Wuhan University (approval number: 2019021), and the signatures of informed consent were obtained from all patients. In order to protect patient confidentiality, we have settled strict procedures to ensure the privacy and anonymity of the participants and excluded their identification before data analysis. We only collected the data we need to meet the research objectives and ensure that data is kept securely.

### 2.2 Data sources

Microarray data of GSE38792 was obtained from the GEO database [GPL6244 platform, Affymetrix Human Gene 1.0 ST Array (transcript gene version), last accessed on 22 January 2023, https://www.ncbi.nlm.nih.gov/geo/query/acc.cgi?acc=GSE38792]. The datasets contain eighteen VAT samples, including ten samples from OSA patients and eight from control patients. The immune-related gene sets (IRGs) were obtained from the IMMPORT database (https://www.immport.org/resources,last accessed on 22 January 2023).

### 2.3 Identification of immune-related differentially expressed genes

We analysis the differentially expressed genes (DEGs) through a “limma” package in R software (Ritchie et al., 2015). A p-value <0.05 was the threshold for identifying DEGs. The Venn online tool (http://bioinformatics.psb.ugent.be/webtools/Venn/) was used to identify IRDEGs, namely the intersection part between DEGs and IRGs.

### 2.4 Construction protein-protein interaction network

After determining the IRDEGs between OSA and control patients, we used the STRING database (https://string-db.org/, version 11.0) to construct the PPI network, the default confidence threshold was 0.4 (Szklarczyk et al., 2017). Then the network was exported to the Cytoscape software (version 3.8.0) for visualization (Shannon et al., 2003). Next, the MCODE plugin (version 2.0.0) in Cytoscape was adopted to find functional gene clusters in the PPI network (Bader and Hogue 2003). The cluster finding parameters were system default. The modules with the highest established score were screened out, and all the genes in this module were identified as the hub genes.

### 2.5 Functional enrichment analyses of hub genes

To further analyze the biological processes and pathways of the hub genes, gene ontology (GO) and the Kyoto Encyclopedia of Genes and Genomes (KEGG) pathway were performed. A free online platform was applied for the functional enrichment analysis and visualization (https://www.bioinformatics.com.cn; last accessed on 22 January 2023). The powerful enrichment criteria were a p-value <0.05.

### 2.6 Analysis of immune cell infiltration

CIBERSORT is a deconvolution algorithm to evaluate the proportions of immune cell subtypes that has been tested for RNA (Ribonucleic Acid) sequencing measurements in Gene expression profiling (Newman et al., 2015). We used the CIBERSORT package to analysis the immune cell infiltration based on the formatted gene expression matrix data in VAT samples. Principal component analysis (PCA) was conducted to determine the difference between OSA and control samples. The different immune infiltration levels of each immune cell between the two groups were analyzed by the “vioplot” package in R version 4.1.1 (https://github.com/TomKellyGenetics/vioplot), a p-value <0.05 was thought to be statistically difference.

### 2.7 Correlation analysis between hub genes and immune infiltration cells

Spearman’s correlation analysis was adopted to evaluate the relationship between hub genes and immune cells. The analysis process and result visualization were conducted by online platform (https://www.xiantao.love; http://www.bioinformatics.com.cn; last accessed on 22 January 2023).

### 2.8 Construction transcription factors regulated network and target drugs analysis

To reveal the potential mechanism of the fat immune dysfunction in OSA patients, we predicted transcription factors (TFs)-target gene pairs among the hub genes using the iRegulon plugin in Cytoscape (Janky et al., 2014). The TF-target pairs from Track rankings database with normalized enrichment score (NES) > 5 was selected. With the correlation results between immune-related hub genes and immune cells, we constructed a TF-mRNA-immune cells regulated network. Besides, we predicted target drugs through the Drug-Gene Interaction Database (DGIdb) (Cotto et al., 2018). The account of hub genes and selected predicted TFs were uploaded to DGIdb to find potential drugs as therapeutic targets of OSA. The results were visualized by https://www.bioinformatics.com.cn (last accessed on 22 January 2023).

### 2.9 Study population and clinical sample collection

To external validate the findings from dataset analysis, a total of 20 obese patients were recruited from our Bariatric and Metabolic Disease Surgery Center, Zhongnan Hospital of Wuhan University, Wuhan, China, from June 2020 to February 2021. VAT samples (omental adipose tissues) were harvested during bariatric surgery. All patients underwent preoperative overnight PSG to screen OSA. The presence of OSA was diagnosed by apnea-hypopnea index (AHI). Briefly, the diagnose criteria was AHI ≥5/h. Of the 20 obese patients, ten patients with AHI ≥5/h were divided into the OSA group, and the rest with AHI <5/h were regard as the control group. Gender, age, and body mass index were matched between the two groups. The clinical characteristics of 20 obese patients can be seen in Table 1.

**TABLE 1:** The basic clinical characteristics of OSA patients and controls.

Variables	Control (*n* = 10)	OSA (*n* = 10)	p-value
Gender(female)	10	10	-
Ages (years)	25 ± 9	30 ± 7	0.229
BMI (kg/m^2^)	31.6 ± 3.7	32.9 ± 2.7	0.381
AHI (events/h)	3.1 ± 1.1	33.8 ± 22.1	<0.001
Comorbidity			
NAFLD	8	8	
Hypertension	0	1	
T2D	0	3	
Hyperlipidemia	2	5	
Hyperuricemia	8	5	

Abbreviations: OSA, obstructive sleep apnea; BMI, body mass index; AHI, apnea hypopnea index; NAFLD, non-alcoholic fatty liver disease; T2D, type 2 diabetes.

### 2.10 RNA extraction

Total RNA from the VAT samples was extracted with TRIzol reagent (15596-026, Invitrogen, America) following a modified isolation protocol (Roy et al., 2020). Briefly, weight a maximum of 500 mg of pure VAT sample and add 1 ml TRIzol accordingly. Homogenise them and keep the tubes on ice. After centrifuge the homogenate, carefully remove the fat layer by pipetting. Add 200 µL of chloroform for phase separation, transfer the upper aqueous layer to another new tube, add 500 µL of isopropyl alcohol for RNA precipitation. Discard the supernatant and add 1 ml of 75% ethanol twice for RNA wash. At last, air-dry the RNA pallet and dissolve it with 30 µL DEPC-treated (Diethypyrocarbonate) water. Quantify RNA concentration and purity was detected by an OD (Optical density) at 260 nm and 280 nm using a Spectrophotometer (NanoDrop One, Thermo Fisher Scientific, Inc.). RNA integrity was examined by electrophoresis in a 1% agarose gel.

### 2.11 Quantitative real-time polymerase chain reaction analysis

cDNA (complementary Deoxyribo Nucleic Acid) was reverse transcribed using All-in-one RT Supermix Kit (R333-01, Vazyme, Nanjing, China) following the manufacturer’s instructions, and RT-qPCR (Real-time quantitative polymerase chain reaction) was performed in triplicate using ChamQ SYBR qPCR Master Mix Kit (Q311-02, Vazyme, Nanjing, China), including the analysis of identified hub gene and macrophage marker gene expression. The thermal cycle profile was as follows: an initial activation was 30 s at 95°C, followed by 40 cycles of denaturation (10 s at 95 °C), annealing (30 s at 60°C) and extension (15 s at 95°C). PCR products were evaluated by melting curve analysis for their specificity and identity. The sequences of primers are available in Supplementary Table S1. The relative expression levels of mRNA (messenger Ribonucleic Acid) were calculated using the 2^−ΔΔ^Ct method with the normalization to the reference gene of *ACTB (Actin Beta)*.

### 2.12 Enzyme-linked immunosorbent assay analysis

Total protein from the VAT samples was extracted using RIPA buffer (P0013B, Beyotime, Beijing, China), and the concentrations of protein were detected using a BCA assay (P0009, Beyotime, Beijing, China). A total of 45 µg protein was used and performed with sample diluent to make up the volume to 100 µL for each ELISA kit. The protein expression levels of three genes (*CRP, sCD40L*, and *GZMB*) were analyzed in triplicate using QuantiCyto Human ELISA kits (Cat#: EHC011 for *CRP*, EHC118 for *sCD40L*, EHC117 for *GZMB*, Neobioscience, Shenzhen, China) following the manufacturer’s instructions. Briefly, Standard and sample general dilution was added to blank wells, and samples or different concentrations of protein standard (100 µL/well) were added to the other corresponding wells. The reaction wells then incubated at 37°C in the dark for 90 min. After washing the plate 5 times, biotinylated antibody diluent was added to blank Wells, and biotinylated antibody working solution (1:30 dilution, 100 µL/well) was added to the remaining Wells. The reaction wells then incubated at 37 °C in the dark for 60 min. After washing the plate 5 times again, dilution of enzyme conjugate was added to blank wells and working solution of enzyme conjugate (1:30 dilution, 100 µL/well) was added to the remaining wells. The reaction wells then incubated at 37 °C in the dark for 30 min. After washing the plate another 5 times, chromogenic substrate (TMB) 100 µL/well was added and incubated at 37°C for 15 min in the dark. The reaction termination solution 100 µL/well was added, and the OD 450 nm value was measured immediately after mixing (within 3 min). Save and record the readings on the instrument.

### 2.13 Immunohistochemistry analysis

VAT samples were fixed in formalin, embedded in paraffin, and cut into 5-µm-thick sections, and then stained for immune-histochemical detection of macrophage polarization markers and interested protein. Briefly, the paraffin sections were deparaffinized and rehydrated with graded alcohols after being incubated at 65°C for 20 min. Antigen retrieval was performed by microwave heating in pH 6.0 Sodium citrate solution. The sections were incubated in 3% H_2_O_2_ for 10 min and blocked in 5% bovine serum albumin (BSA) for 30 min at room temperature. Then followed by incubation with primary antibodies overnight at 4°C and secondary antibody for 30 min at room temperature. Negative controls were performed by replacing the primary antibody with phosphate buffer solution (PBS). The use of Diaminobenzidine (DAB) as a chromogen was to visualize positive cells. Primary antibodies *CD11c* (1:400 dilution, 17342-1-AP, Proteintech Group, China) and *CD206* (1:500 dilution, ab64693, Abcam,United States) were used to identify M1 and M2 macrophages, respectively (Julla et al., 2019; Zhou et al., 2020; Galarraga-Vinueza et al., 2021). Primary antibodies *CCL20/MIP3α* (1:100 dilution, DF2238, Affinity, China) was also used for immunohistochemistry (IHC) analysis. The target protein expression was evaluated by integrated optical density (IOD)/area assay through ImageJ software.

### 2.14 Statistical analysis

Public dataset analysis was completed in R software (version 4.0.1) and online platform, experimental data were analyzed with GraphPad Prism 8 (Graph-Pad Software, CA, United States). Values were presented as mean ± SD (Standard Deviation). The student’s t-test was adopted to analyze the two independent groups regarding gene and protein expression levels, and the statistically significant criteria was a p-value <0.05.

## 3 Results

### 3.1 Identification of immune-related differentially expressed genes

**FIGURE 1:**
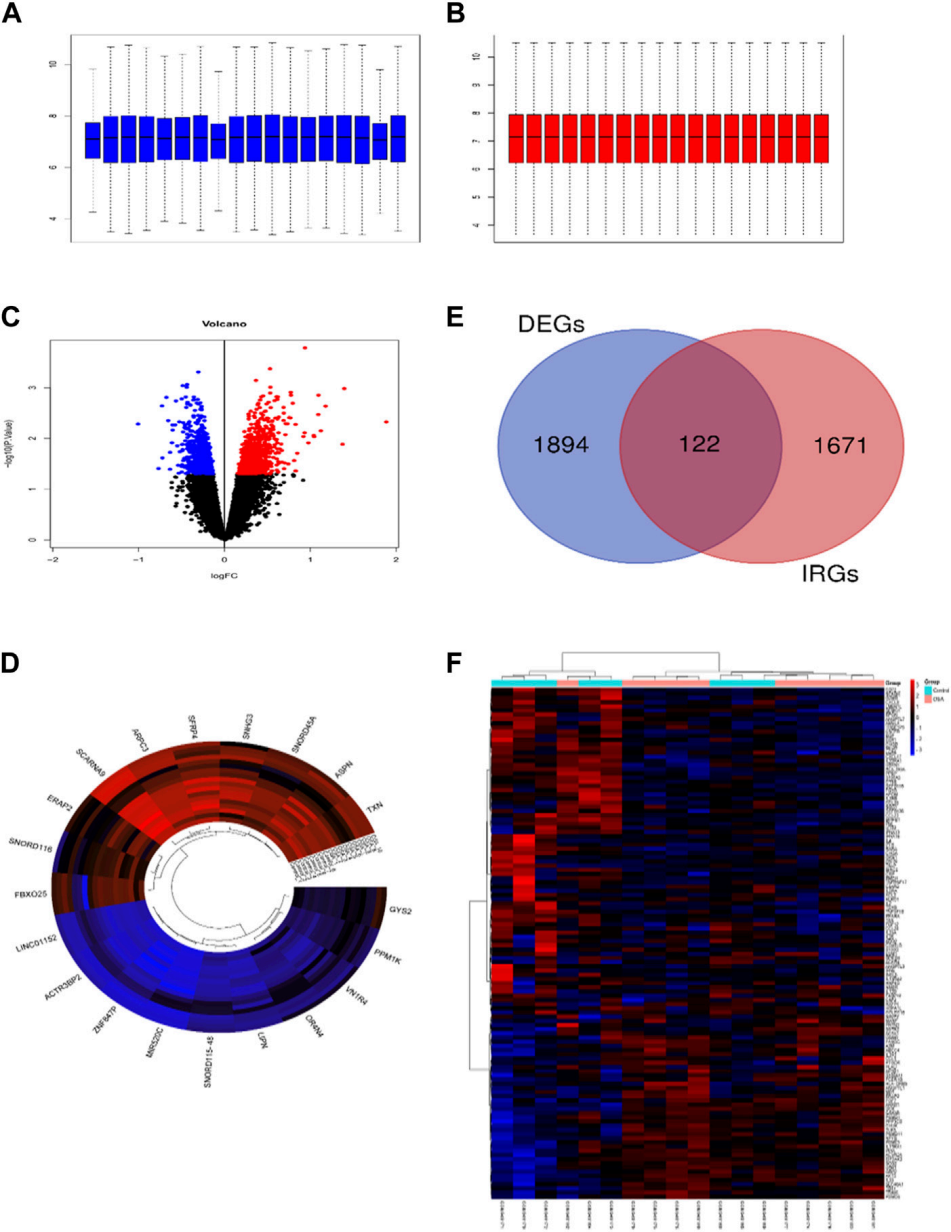
Identification of immune-related differentially expressed genes (IRDEGs). Box plots show the distribution of the relative gene expression before **(A)** and after **(B)** normalization of GSE38792. Each box corresponds to one sample. The middle line corresponds to the median. **(C)** Volcano plot of differentially expressed genes (DEGs). DEGs were screened with the criteria of p-value <0.05. **(D)** The cluster circular heat map showing the top 10 upregulated and downregulated DEGs. **(E)** Venn diagram showing the intersection of DEGs and immune-related genes (IRGs). **(F)** The heatmap of 122 IRDEGs.

The raw microarray data from GSE38792 was normalized by the RMA (Robust Multi-Array Average) method to eliminate batch expression difference (Figures 1A, B). Then we identified a total of 2016 unique DEGs in the VAT of OSA patients compared with normal controls by the screening threshold of *p* < 0.05. Figures 1C, D showed the volcano and heatmap plots of DEGs. Figure 1E displayed the Venn diagram of 122 IRDEGs. The heatmap of 122 IRDEGs was shown in Figure 1F, including 44 down-regulated genes and 78 up-regulated genes.

### 3.2 Protein-protein interaction network

**FIGURE 2:**
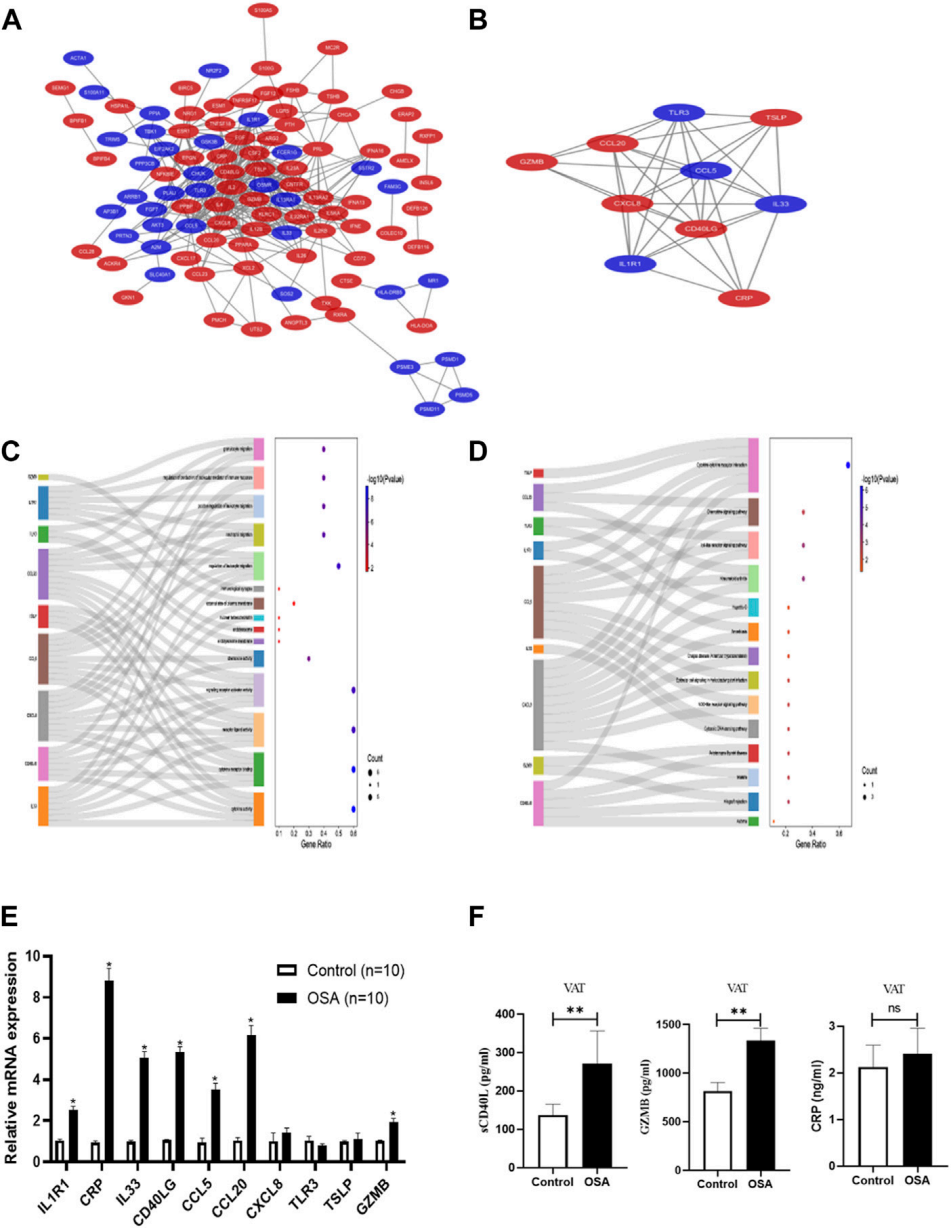
Protein-protein interaction (PPI) network construction, hub gene identification and functional enrichment analysis. **(A)** The PPI network based on STRING database and Cytoscape software, red color represents upregulated genes and blue color represent downregulated genes. **(B)** Hub genes identified by Cytoscape MCODE plug-in, red color represents upregulated genes and blue color represent downregulated genes. **(C)** Sankey dot of GO enrichment analyses of hub genes. **(D)** Sankey dot of KEGG pathway enrichment analyses of hub genes. The dot plot showed the hub genes specific to GO terms or KEGG pathways and the total number of genes in each enriched pathway. External validation of the hub genes at mRNA level **(E)** and protein level **(F)**.

PPI network comprising 103 nodes and 354 edges was constructed using the STRING database to investigate the underlying biological functions of IRDEGs (Figure 2A). Through the MCODE plugin in Cytoscape, we found the most significant module (Score = 8.222) in the PPI network of 122 IRDEGs, comprising 10 immune-related hub genes (*TLR3*, *IL33, GZMB, IL1R1, CRP, CXCL8, CCL5, TSLP, CCL20, and CD40LG*) (Figure 2B). The functional annotation of these genes was provided by GeneCards (https://www.genecards.org/) in Supplementary Table S2.

### 3.3 Functional enrichment analysis of hub genes

GO and KEGG analysis were performed to explore the biological processes and pathways of the immune-related hub genes. They mainly involved in cytokine activity, chemokine activity, CCR chemokine receptor blinding, G protein-coupled receptor binding, and phospholipase activator activity from the results of GO analysis (Figure 2C; Supplementary Table S3). The significantly enriched pathways were cytokine-cytokine receptor interaction, rheumatoid arthritis, toll-like receptor signaling pathway, chemokine signaling pathway, and nod-like receptor signaling pathway as revealed by KEGG analysis results (Figure 2D; Supplementary Table S4).

### 3.4 External validation for the immune-related hub genes in collected clinical samples

Through seeking multidisciplinary collaboration, we successfully collected VAT samples to validate the immune-related hub genes from OSA and control patients. The relative mRNA expression level of the 10 immune-related hub genes were shown in Figure 2E. The levels of *CRP, CD40LG, CCL20, GZMB, IL1R1, IL33, and CCL5* in VAT of OSA group were significantly up-regulated, while only the relative mRNA expression of *CRP, CD40LG, CCL20 and GZMB* were consistent with the bioinformatics analysis results. As proteins are the executors of gene function, we subsequently analyzed the protein expression level of *CRP, CD40LG, CCL20 and GZMB,* as shown in Figure 2F. The *sCD40L* and *GZMB* levels were higher in the VAT of OSA patients (270.71 ± 85.37 vs. 136.99 ± 28.27 pg/ml, *p* = 0.011; 1,337.68 ± 394.55 vs. 814.95 ± 278.33 pg/ml, *p* = 0.017), which were consistent with the relative mRNA expression levels. The *CRP* levels in VAT showed the same tendency in OSA patients (2.41 ± 0.55 vs. 2.13 ± 0.46 ng/ml), but no statistically significant (*p* = 0.369) was found. While the protein expression levels of *CCL20* in IHC showed no difference (Supplementary Figure S1).

### 3.5 Immune infiltration analysis

**FIGURE 3:**
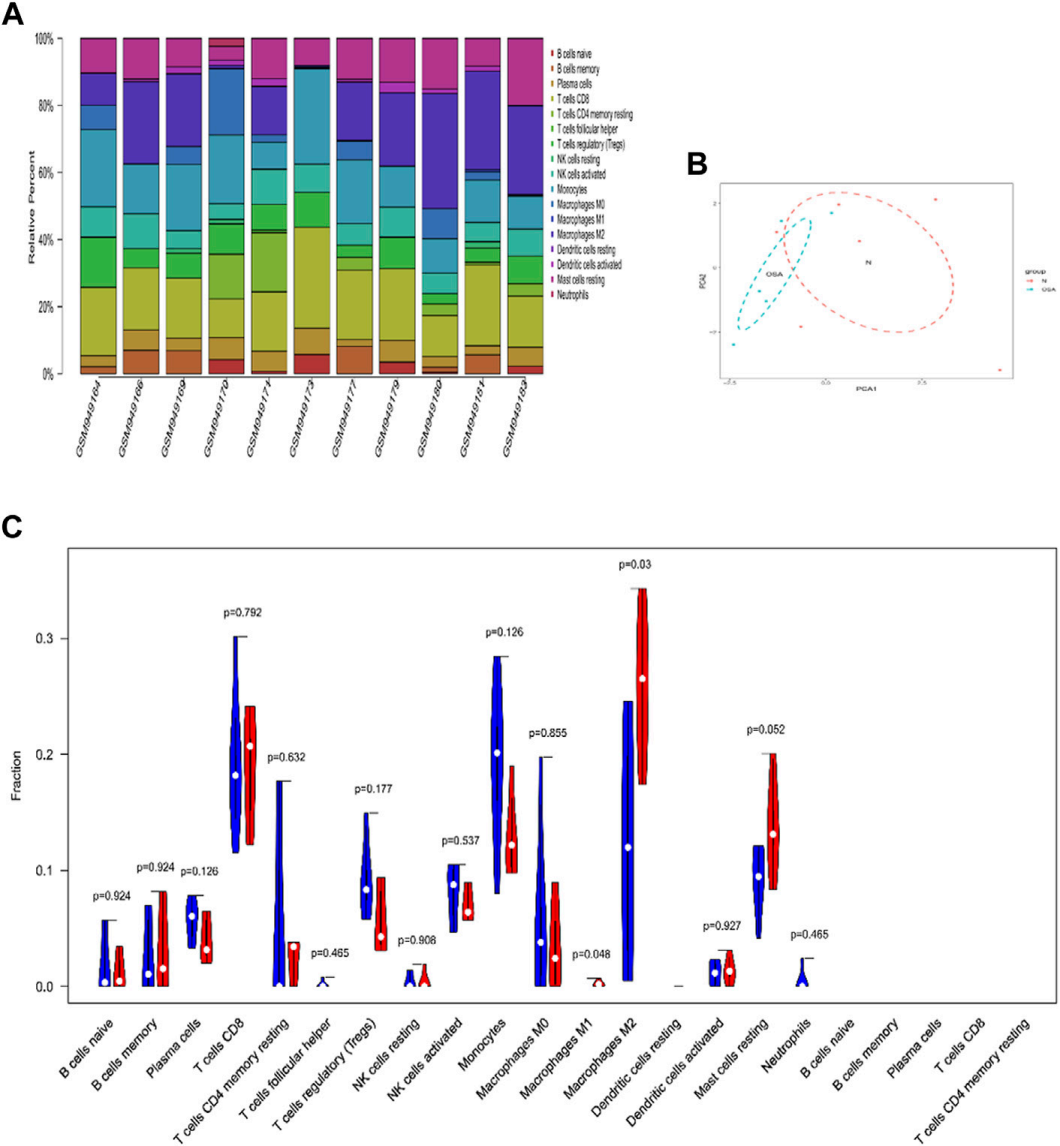
The landscape of immune infiltration in VAT between OSA and controls. **(A)** The relative percentage of 22 subpopulations of immune cells in 11 samples from GSE38792 datasets. **(B)** Principal components analysis performed on all samples. **(C)** Violin plot of differences in 22 infiltrating immune cells between OSA and normal controls. The normal group was marked as blue color and OSA group was marked as red color. *p* values <0.05 were considered as statistical significance.

Immune infiltration analysis was performed between OSA and control patients. Figure 3A displayed the relative proportion of immune cell subtypes after filtering out the samples with *p* < 0.05. In VAT, monocytes and macrophages accounted for the highest proportion of immune cell types, followed by CD8 T cells. The PCA results as displayed in Figure 3B showed the group-bias clustering among the groups. Compared with control patients, OSA patients were characterized by macrophage infiltration and contained a higher proportion of M1 and M2 macrophages in VAT (*p* < 0.05, respectively) (Figure 3C).

### 3.6 Analysis of the characteristics of macrophage infiltration in collected clinical samples

**FIGURE 4:**
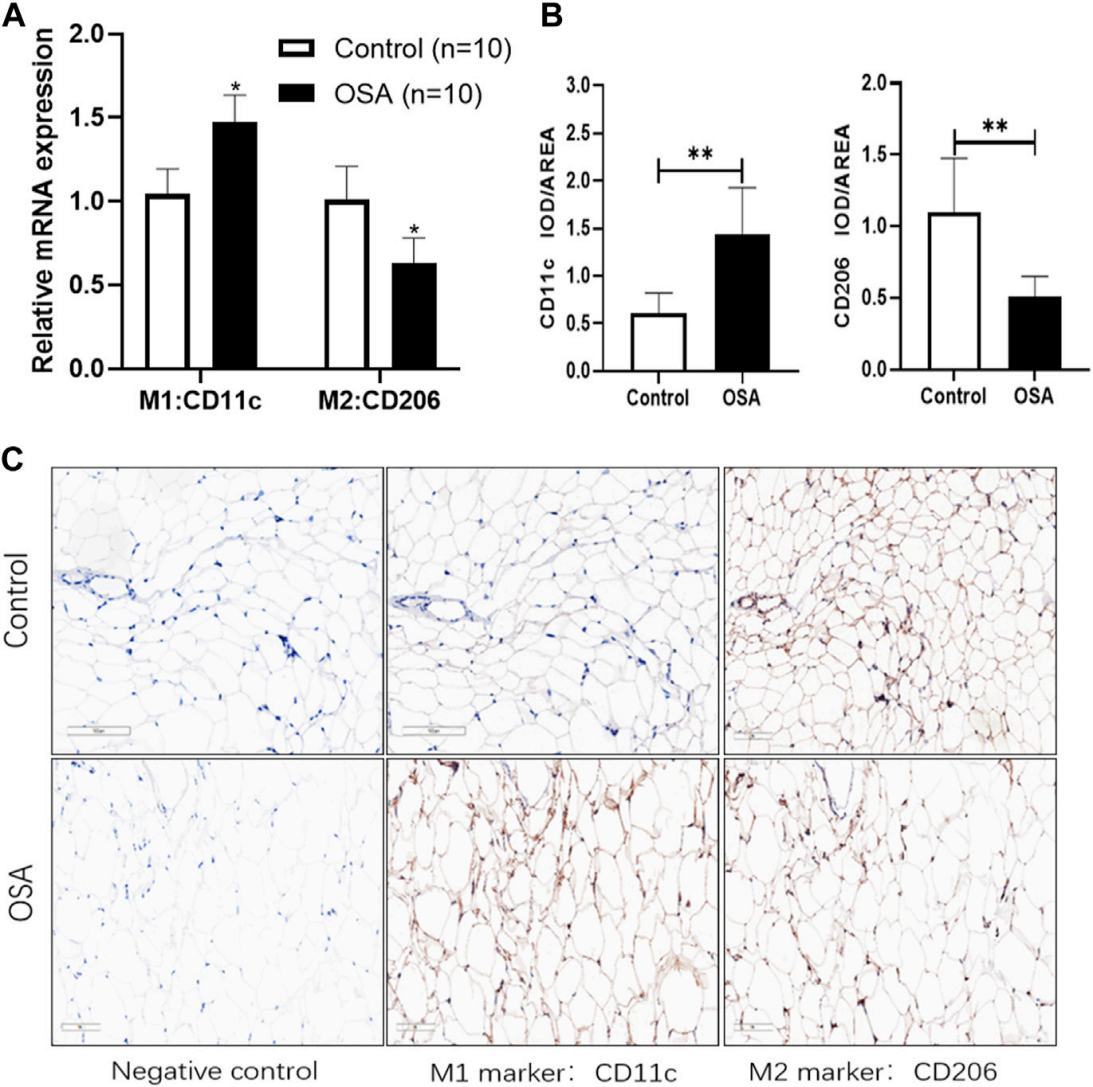
Analysis of the characteristics of macrophage infiltration in collected clinical samples. **(A)** The relative mRNA expression level of macrophage markers by RT-qPCR methods. **(B)** The protein expression level of macrophage markers by immunohistochemistry methods. The target protein expression was evaluated by integrated optical density (IOD)/area assay through ImageJ. **(C)** Representative immunohistochemistry images from the same plane. Magnification, ×200, scale bar = 100 μm. Data are presented as the mean ± SD (*n* = 10), **p < 0.01.

External validation of human VAT macrophage infiltration characteristics was also performed. The relative mRNA expression level of M1 marker *CD11c* was increased by nearly 0.5-fold change while M2 markers *CD206* was decreased by 0.4-fold change in the OSA group when compared to the control group (Figure 4A). The protein expression level of human VAT macrophage markers further revealed that obese OSA patients were characterized by significantly higher protein expression levels of M1 macrophages (*CD11c*,1.43 ± 0.50 vs. 0.61 ± 0.21 IOD/AREA, *p* < 0.001), while a lower M2 macrophages (*CD206*, 0.51 ± 0.15 vs. 1.10 ± 0.38 IOD/AREA, *p* < 0.001) in VAT (Figures 4B, C). Compared to control patients, the presence of OSA accelerated the conversion of VAT macrophages to pro-inflammatory phenotype in obese patients.

### 3.7 Correlation analysis between hub genes and immune infiltration cells

**FIGURE 5:**
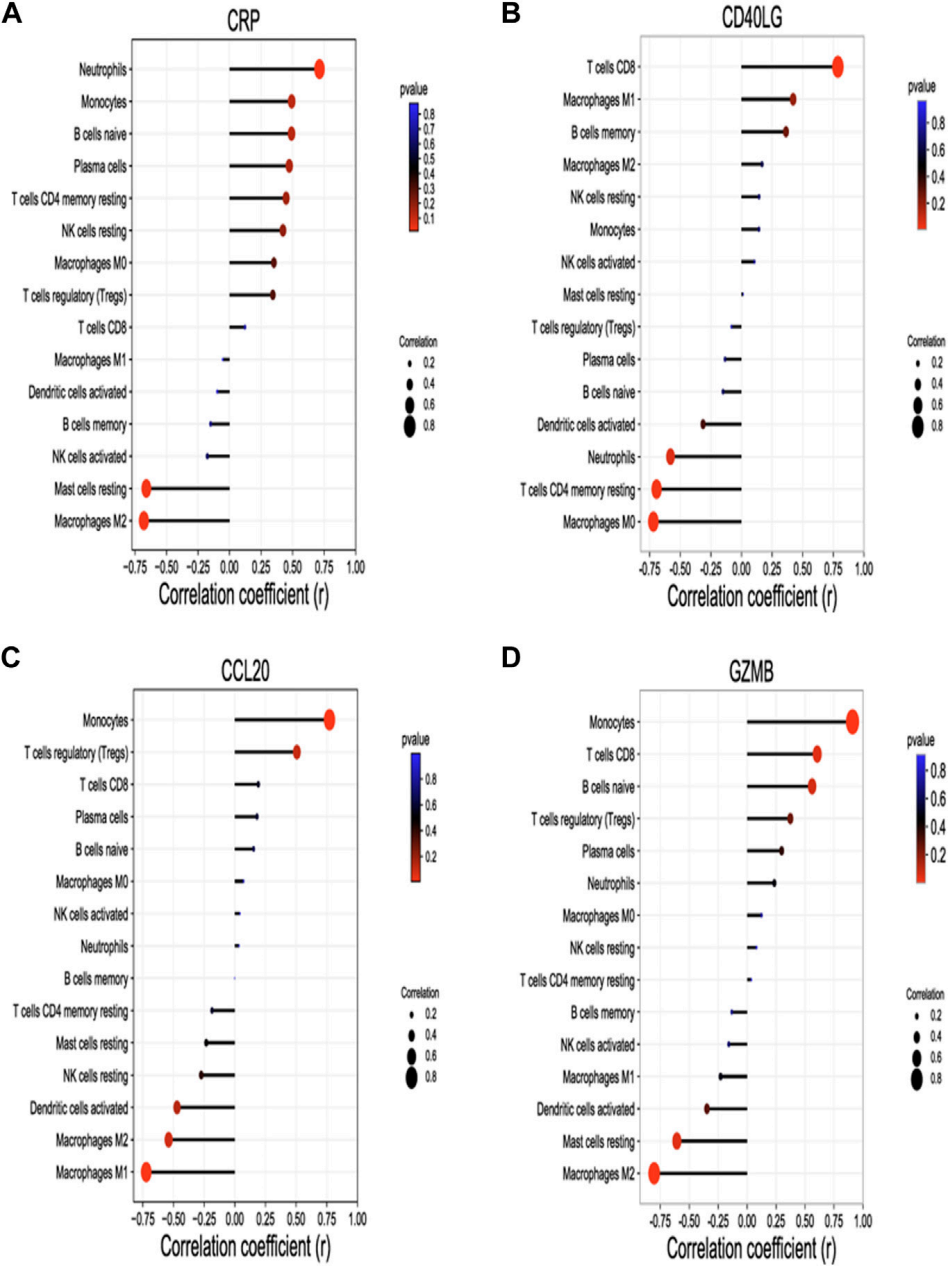
Correlation between hub genes and Immune infiltration cells. Spearman’s correlation analysis between *CRP* **(A)**, *CD40LG* **(B)**, *CCL20* **(C)**, *GZMB* **(D)** and infiltrating immune cells, respectively.The four hub genes were validated by RT-qPCR. The size of the dots represents the strength of the correlation between genes and immune cells; the larger the dots, the stronger the correlation. The color of the dots represents the p-value, the redder the color, the lower the p-value. *p* < 0.05 was considered statistically significant. *CRP*, C-Reactive Protein; *CCL20*, C-C Motif Chemokine Ligand 20; *CD40LG*, CD40 Ligand; *GZMB*, Granzyme B.

Significant correlation between four identified hub genes and immune infiltration cells were shown in Figure 5. *CRP* was positively correlated with neutrophils (r = 0.71, *p* = 0.014) and negatively correlated with M2 macrophages (r = -0.68, *p* = 0.021), and mast cells resting (r = -0.66, *p* = 0.027); *CD40LG* was positively correlated with CD8 T cells (r = 0.79, *p* = 0.004) and negatively correlated with CD4 memory resting T cells (r = -0.69, *p* = 0.018) and M0 macrophages (r = -0.72, *p* = 0.013); *CCL20* was positively correlated with monocytes (r = 0.77, *p* = 0.005) and negatively correlated with M1 macrophages (r = -0.72, *p* = 0.012); *GZMB* was positively correlated with monocytes (r = 0.91, *p* ≤ 0.001), CD8 T cells (r = 0.61, *p* = 0.049) and negatively correlated with M2 macrophages (r = -0.80, *p* = 0.003), and mast cells resting (r = -0.61, *p* = 0.048).

### 3.8 The transcription factors regulated network and target drugs in OSA patients

**FIGURE 6:**
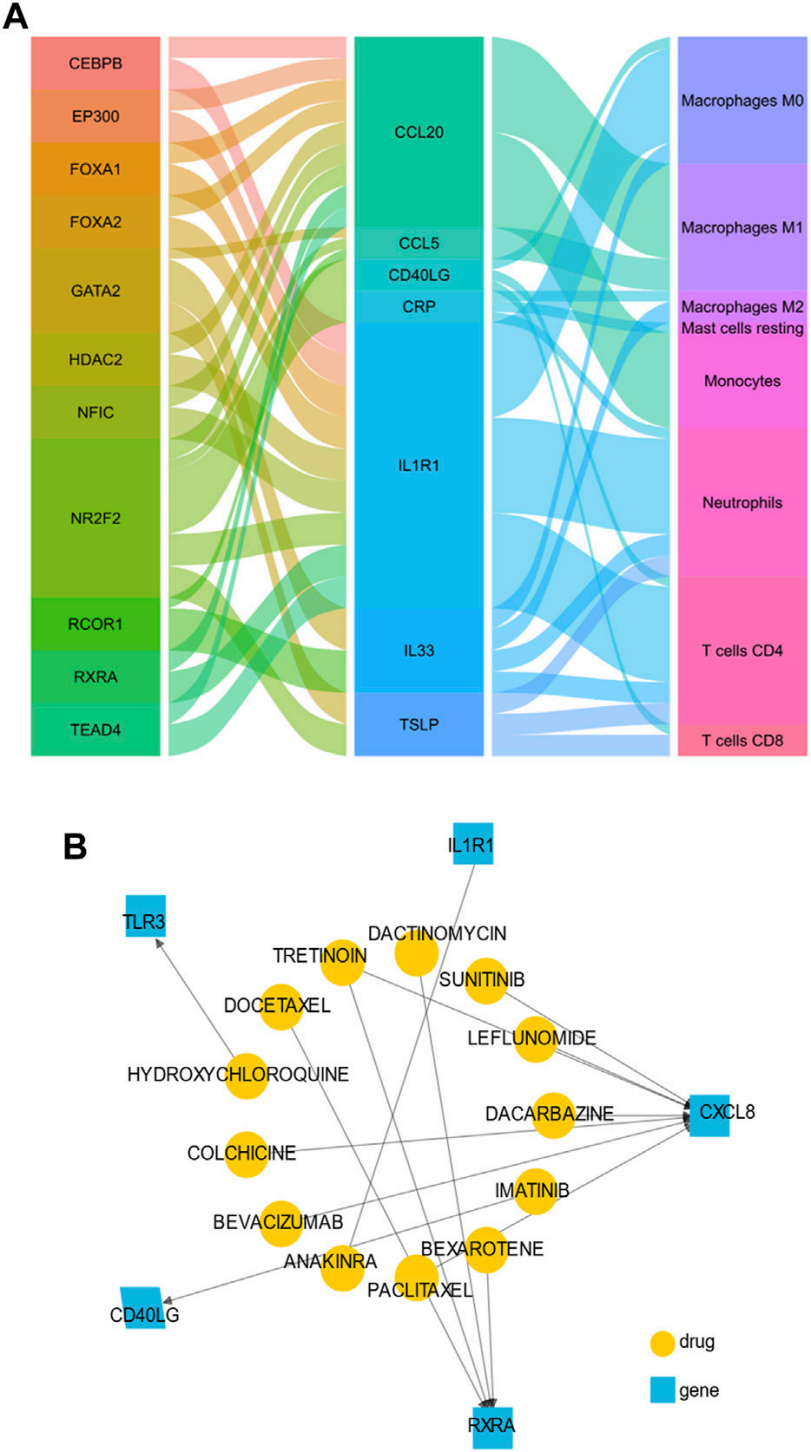
The transcription factors (TFs) regulated network and target drugs in OSA patients. **(A)** The alluvial plot showing the regulatory network of TFs-genes-immune cells. The left column represents predicted TFs, the middle column represents immune-related hub genes, the right column represents immune cells, and the edge represents the relationship between them. A larger edge width indicates the number of TFs and immune cells **(B)** Drug-gene network using drug-centric fashions. Yellow circles indicate predictive drug, and blue squares indicate immune-related hub genes.

We obtained 11 TFs (*NR2F2, FOXA1, NFIC, HDAC2, EP300, TEAD4, CEBPB, GATA2, RCOR1, FOXA2, and RXRA*) and 27 TF-target pairs (Supplementary Table S5). The statistically significant correlation immune cells (r > 0.5 and *p* < 0.05) were monocytes, M0 macrophages, M1macrophages, M2 macrophages, neutrophils, resting mast cells, CD4 memory resting T cells and CD8 T cells. Interestingly, *RXRA* and *NR2F2* were also among 122 IRDEGs, indicating that the two TFs play a key role in the potential mechanism of VAT homeostasis of OSA patients. The 27 TF-target pairs and the relationship between target genes and immune cells were demonstrated in Figure 6A. Through the DGIdb database, we also found several potential drugs that target hub genes and TFs, Figure 6B showed a visualization plot of drug-gene network.

## 4 Discussion

Given the strong link between OSA, obesity and their related comorbidity, adipose tissue may act as a key player in the pathogenesis and progress of OSA (Ryan et al., 2019). Compared to subcutaneous adipose tissue, VAT secretes more hormones and proinflammatory cytokines that induce metabolic dysfunction, due to VAT hormones preferential enter the portal circulation and directly alter glucolipid metabolism by the liver (Liu and O'Byrne 2020). Therefore, we initially focus on the visceral adipose tissue (VAT) homeostasis of OSA patients through a multidisciplinary approach. In addition, Gharib et al. (2013) found that “Immunity and Inflammation” were the most upregulated modules in the VAT of OSA patients, but little is known about the key genes and their relationship with immune cell infiltration in this module. Herein, we performed the dataset analysis to explore the effect of immune-related genes and immune infiltration characteristics on OSA-related metabolic dysregulation. To our knowledge, this is the first study that collects clinical VAT samples to validate the findings from bioinformatics analysis, which provides direct evidence for adipose tissue as a proinflammatory target organ for OSA-associated metabolic complications. Furthermore, our study found the biological function of *CD40LG* and *GZMB* might be important for the VAT homeostasis of OSA patients, those two immune-related genes were first reported in the VAT of OSA patients, and their interaction with macrophages and involved pathways might provide new insights for understanding molecular mechanisms of OSA-related metabolic dysregulation.

In the present study, we reanalyzed the only visceral fat transcriptome dataset GSE38792 including OSA patients and identified a total of 122 IRDEGs. Then we used the 122 IRDEGs to construct a PPI network and found 10 hub immune-related genes, including *IL1R1, CRP, IL33, CD40LG, CCL5, CCL20, CXCL8, TLR3, TSLP*, and *GZMB*. After an initial validation by RT-qPCR, the relative mRNA expression of four hub genes (*CRP, CD40LG, CCL20, and GZMB*) was consistent with the bioinformatics results, while three genes (*IL1R1, IL33, and CCL5*) showed the opposite results, and the rest three genes (*CXCL8, TLR3, and TSLP*) showed no significant difference. Interestingly, the seven validated hub genes played a pro-inflammatory role when the relative mRNA expression level increased. We further validated the protein expression level of the four hub genes (*CRP, CD40LG, CCL20, and GZMB*) by ELISA and IHC methods. Finally, *CD40LG* and *GZMB* were verified to be consistent with the bioinformatics results regardless of the mRNA and protein expression levels. Hence, the results of the bioinformatic analysis are not always reliable, especially in small sample datasets, experimental validation is necessary to increase confidence. In addition, we did not further establish an immune-related diagnostic model for OSA when compared to previous studies (Li et al., 2017; Gu et al., 2019; Cao et al., 2021; Peng et al., 2021; Liu et al., 2022), because of the invasive procedures for harvesting VAT.

*CD40LG*, which binds *CD40* and triggers pro-inflammatory mediators on the surface of various cell types, was also found to increase in children with OSA and decreased after adenotonsillectomy (Gozal et al., 2007). Soluble CD40 ligand (*sCD40L*) can be a marker for endothelium-related activation and a variety of cardiovascular disorders (Mach et al., 1998; Lutgens & Daemen 2002; Lobbes et al., 2006). Adult male patients with moderate to severe OSA also had significantly higher serum *sCD40L* levels than obese control subjects and nasal continuous positive airway pressure significantly decreased serum levels of *sCD40L* (Minoguchi et al., 2007). Previous studies have only examined serum *sCD40L* levels in OSA patients, and we detected the protein expression of *sCD40L* in the VAT for the first time. Our GO analysis results revealed that *CD40LG* was mainly involved in receptor-ligand activity, chemokine activity, cytokine activity, signaling receptor activator activity and regulation of production of molecular mediator of immune response. With regard to KEGG pathway analysis, *CD40LG* was mainly involved in Cytokine-cytokine receptor interaction pathway. Notably, *CD40LG* in adipose tissue was mainly involved in the progression of OSA by regulating cytokine interaction. *GZMB* belongs to the granzyme subfamily of proteins and is involved in the signaling pathways of apoptosis, necrosis, and inflammation. Mahzad Akbarpour found that the low *GZMB* levels in intratumoral CD8^+^ T cells under tumor microenvironment contributes to the maintenance of self-renew ability of cancer stem cells, which might explain the poorer outcomes of the presence of OSA in cancer patients (Akbarpour et al., 2017). To date, rare studies have reported the expression of *GZMB* in VAT, and our study showed that *GZMB* was upregulated in VAT of OSA patients, but the role of *GZMB* in the occurrence and development of OSA-related morbidity needs further studied. In addition, the correlation between OSA and elevated *CRP* levels has been reported (Gozal et al., 2012; Tie et al., 2016). Although our results showed no statistical difference, the relative expression of *CRP* in VAT was slightly elevated. White fat inflammation was a major contributor to increased *CRP* in obesity, and OSA should be taken into consideration to explain the high *CRP* levels in obese patients (Paepegaey et al., 2015). In summary, the biological functions and involved signaling pathways of *CD40LG* and *GZMB* indicated their important roles in immunity and inflammation modules in the VAT of OSA patients, which may broaden the knowledge of previous findings.

The biological functions of *CD40LG* and *GZMB* were associated with immune cells, so we performed adipose tissue immune infiltration analysis between OSA and control patients. Our data demonstrated the immune cell changes of VAT in the OSA group. Consistent with previous findings (Peng et al., 2021), monocyte-macrophages accounted for the highest proportion of immune cell types, followed by CD8 T cells. The changes in M1 and M2 macrophage proportion showed a significant difference between OSA and the control group. Macrophages are mainly involved in inflammatory responses and microbial killing and their role in the adipose tissue immune microenvironment that induces the pro-inflammatory M1 phenotype and subsequent insulin resistance has been reported in rodent experiments (Murphy et al., 2017; Ryan 2017). Similarly, chronic intermittent hypoxia in OSA-induced adipose tissue macrophage inflammation contributes to dyslipidemia and atherogenesis (Poulain et al., 2014). Then we validated the results using RT-PCR and IHC methods and found that macrophage infiltration, especially pro-inflammatory M1 phenotype in VAT, was a hallmark feature in OSA patients independently of obesity. The presence of OSA exacerbates macrophage infiltration in adipose tissue and is metabolically dysfunctional in obese patients. Our results favor macrophages and inflammation are involved in OSA-related metabolic dysfunction, CD11c-labeled proinflammatory macrophage may be the predominant macrophage subset in VAT of OSA patients, which provides direct evidence for adipose tissue as a proinflammatory target organ for OSA-associated metabolic complications.

By analyzing the correlation between the validated hub genes and immune cells, we found that the expression of *CD40LG* was positively correlated with CD8 T cells and negatively correlated with M0 macrophages and memory resting CD4 T cells. *CD40LG* is expressed on the surface of T cells, and CD8 T cells are crucial members of adaptive immune response (Stelzer et al., 2016). The interaction between T cells and macrophages may be induced by *CD40LG. GZMB* was positively correlated with monocytes and CD8 T cells and negatively correlated with M2 macrophages and resting mast cells. *GZMB* is generally secreted by cytotoxic T lymphocytes and induces target cell apoptosis (Stelzer et al., 2016). The negatively correlation with M2 macrophages hinted that *GZMB* might occur in the early stage of adipose tissue inflammation. *CRP* was positively correlated with neutrophils and negatively correlated with M2 macrophages and resting mast cells. *CCL20* was significantly positively correlated with monocytes, and negatively correlated with M1 macrophages. Studies have shown that adipose-resident macrophage numbers are positively related to circulating inflammatory markers such as *CRP* and *TNFα* (*CD40LG* belongs to *TNF* family members), and adipose inflammation is thought to be the main source of systemic inflammation and metabolic disorder associated with obesity (Paepegaey et al., 2015; Kunz et al., 2021). Therefore, better understanding of the relationship between immune-related genes and immune infiltration cells may contributes to discovering novel small molecules or other promising candidates as immunotherapies of OSA-associated metabolic complications.

In order to find novel immunotherapies of OSA-associated metabolic complications, we predicted candidate TFs and target drugs for the hub genes by cytoscape software and online database. We discovered two TFs, namely *RXRA* and *NR2F2,* were also belonging to the 122 IRDEGs. *RXRA,* Retinoid X Receptor Alpha*,* is a common binding partner to many other nuclear receptors such as PPARs, vitamin D receptors and liver X receptors. It also promotes myelin debris phagocytosis and remyelination by macrophages (Natrajan et al., 2015). Bexarotene, one of the small molecules predicted for target *RXRA gene,* can improve cholesterol homeostasis and inhibit atherosclerosis progression in a mouse model of mixed dyslipidemia (Lalloyer et al., 2006). *NR2F2,* Nuclear Receptor Subfamily two Group F Member 2*,* is an important regulator of differentiation, which has been linked to tissue homeostasis and its abnormal expression may lead to infertility, aberrant development of the vascular system, and metabolic diseases (Stelzer et al., 2016). Although no drugs were predicted to target *NR2F2 gene* in the DGIdb database, its role in inflammation and immunity cannot be ignored. Imatinib, a protein kinase inhibitor predicted for target *CD40LG* gene, has been reported to ameliorate COVID-19-induced metabolic complications (Li et al., 2022). In summary, the candidate TFs and target drugs for the immune-related hub genes contribute to finding novel immunotherapies of OSA-associated metabolic complications, but more animal and clinical trials are essential for drug efficacy validation and achieving clinical translation.

Nevertheless, our research also has some limitations. Firstly, CIBERSORT is an analytical tool based on limited existing gene expression data that may underestimate the potential heterotypic interactions of cells. We believe that the immune cell characteristics of adipose tissue in OSA patients will be better illumination with the widespread of single-cell sequencing (Acosta et al., 2017). Secondly, the sample size is small both in the training dataset and in external validation, owing to the difficulty of collecting VAT samples that meet the inclusion criterion (eg. overnight PSG completed; gender, age, and body mass index must be matched). Thirdly, we only chose *CD11c* and *CD206* primary antibodies to label M1 and M2 macrophages, respectively. Since there are so many markers for macrophages (Shapouri-Moghaddam et al., 2018), we might have overlooked the role of other macrophage subsets in fat homeostasis of OSA patients. Finally, we only validate the hub genes and macrophage infiltration in the VAT of OSA, further research is needed to comprehensively identify the potential mechanism of each IRDEG (immune-related differentially expressed gene) and their interaction with immune cells in OSA-related cell and mouse models.

## 5 Conclusion

In conclusion, our research found that *CD40LG* and *GZMB* played important roles in immunity and inflammation modules in the VAT of OSA patients, and pro-inflammatory M1 macrophage in VAT was a hallmark feature in OSA patients independently of obesity. The interaction between *CD40LG, GZMB* and adipose tissue macrophages not only provides new insights for understanding molecular mechanisms but also be of great significance in discovering novel small molecules or other promising candidates as immunotherapies of OSA-associated metabolic complications.

## Data Availability

The datasets presented in this study can be found in online repositories. The names of the repository/repositories and accession number(s) can be found in the article/Supplementary Material.

## Supplementary material

Supplementary Figure S1 Representative immunohistochemistry images of CCL20 in VAT between OSA and control patients. OSA, obstructive sleep apnea; VAT, visceral adipose tissue.

Supplementary Figure S2 Representative graph of melting temperature of PCR results.

Supplementary Figure S3 Representative graph of electrophoretic results for examining RNA integrity.

Supplementary Table S1 Sequences of hub genes and markers of macrophages.

Supplementary Table S2 The functional annotation of 10 hub genes.

Supplementary Table S3 GO analyses results of immune-related Hub genes (Top 5 of each category according to P value). “Count” means how many DEGs were involved.

Supplementary Table S4 Kyoto Encyclopedia of Genes and Genomes (KEGG) pathway analysis of immune-related hub genes. (Top 14 according to P value). "Count" means how many DEGs were involved.

Supplementary Table S5 The transcription factors (TFs) regulated network in OSA patients.
