# Supplementary material for: CD40LG and GZMB were correlated with adipose tissue macrophage infiltration and involved in obstructive sleep apnea related metabolic dysregulation: Evidence from bioinformatics analysis
Source: Front Genet. 2023 Feb 27;14:1128139. doi: 10.3389/fgene.2023.1128139 (PMC10009156; doi:10.3389/fgene.2023.1128139)
Supplement: Supplementary file 2 [file Table2.DOCX]

#设置工作目录

setwd("C:\\Users\\Administrator\\Desktop\\GSE38792")

library(limma)

library("impute")

geo_data<-read.table("data.txt",sep="\t",header=T)

geo_data<-as.matrix(geo_data)

rownames(geo_data)=geo_data[,1]

geo_exp<-geo_data[,2:ncol(geo_data)]

dimnames<-list(rownames(geo_exp),colnames(geo_exp))

geo_exp<-matrix(as.numeric(as.matrix(geo_exp)),nrow=nrow(geo_exp),dimnames=dimnames)

mat=impute.knn(geo_exp)

geo_data=mat$data

geo_data=avereps(geo_data)

pdf(file="raw_box.pdf")

boxplot(geo_data,col = "blue",xaxt = "n",outline = F)

dev.off()

geo_data=normalizeBetweenArrays(as.matrix(geo_data))

pdf(file="normal_box.pdf")

boxplot(geo_data,col = "red",xaxt = "n",outline = F)

dev.off()

#进行分组

class <- c(rep("control",8),rep("OSA",10))

#构建矩阵

design <- model.matrix(~0+factor(class))

colnames(design) <- c("control","OSA")

#线性模型拟合

fit <- lmFit(geo_data,design)

cont.matrix<-makeContrasts(OSA-control,levels=design)

#构建对比模型，比较两个实验条件下的表达数据

fit1 <- contrasts.fit(fit, cont.matrix)

#贝叶斯检验

fit1 <- eBayes(fit1)

#保存所有基因的表达数据和统计量

allgene<-topTable(fit1,adjust='fdr',number=100000)

write.table(allgene,"allgene.xls",sep="\t",quote=F)

normaldata<-allgene[order(allgene$logFC),]

normaldata1<-rbind(Gene=colnames(normaldata),normaldata)

write.table(normaldata,"normaldata.txt",sep="\t",quote=F,col.names=F)

#保存差异基因

diffgene <- allgene[with(allgene, (abs(logFC)>=0 & P.Value < 0.05 )), ]

write.table(diffgene,"diffgene.xls",sep="\t",quote=F)

#保存上调差异基因

Upgene <- allgene[with(allgene, (logFC>=0 & P.Value < 0.05 )), ]

write.table(Upgene,"upgene.xls",sep="\t",quote=F)

#保存下调差异基因

Downgene <- allgene[with(allgene, (logFC<=0 & P.Value < 0.05 )), ]

write.table(Downgene,"down.xls",sep="\t",quote=F)

#将差异基因的校正后的表达值保存起来

diffexp=geo_data[rownames(diffgene),]

diffexp1=rbind(id=colnames(diffexp),diffexp)

write.table(diffexp1,"diffexp.txt",sep="\t",quote=F,col.names=F)

#画火山图

inputfile_vol="vol.txt"

mydata<-read.table(inputfile_vol,header=T,row.names=1,check.names=F)

down <- mydata[mydata$logFC <= 0 & mydata$P.Value<0.05,]

up <- mydata[mydata$logFC >= 0 & mydata$P.Val<0.05,]

no <- mydata[(mydata$P.Val>0.05),]

down<- transform(down,P.Value=-log10(down$P.Value))

up<- transform(up,P.Value=-log10(up$P.Value))

no<- transform(no,P.Value=-log10(no$P.Value))

pdf("vol1.pdf")

xm=max(abs(mydata$logFC))

ym=max(-log10(mydata$P.Value))

plot(no$logFC,no$P.Value,xlim = c(-xm,xm),ylim=c(0,ym),col="black",pch=16,cex=0.9,main = "Volcano",xlab = "logFC",ylab="-log10(P.Value)")

points(up$logFC,up$P.Value,col="red",pch=16,cex=0.9)

points(down$logFC,down$P.Value,col="blue",pch=16,cex=0.9)

abline(v=0,lwd=2)

dev.off()

#画热图

inputheatmap="diffexp.txt"

library(pheatmap)

data<-read.table(inputheatmap,sep="\t",header=T,row.names=1,check.names=F)

data<-data[1:20,] #选择前20个基因

pdf("heatmap2.pdf")

pheatmap(data,display_numbers = F,fontsize_row=7,fontsize_col=10,cluster_cols = T,cluster_rows = T,color = colorRampPalette(c("green", "black", "red"))(50))

dev.off()

library("limma")

NORCount=8

OSACount=10

setwd("C:\\Users\\Administrator\\Desktop\\GSE38792\\normalize") #设置工作目录

rt=read.table("sampleExp.txt",sep="\t",header=T,check.names=F) #读取文件

rt=as.matrix(rt)

rownames(rt)=rt[,1]

exp=rt[,2:ncol(rt)]

dimnames=list(rownames(exp),colnames(exp))

data=matrix(as.numeric(as.matrix(exp)),nrow=nrow(exp),dimnames=dimnames)

data=avereps(data)

data=data[rowMeans(data)>0,]

group=c(rep("NOR",NORCount),rep("OSA",OSACount))

design <- model.matrix(~factor(group))

colnames(design)=levels(factor(group))

rownames(design)=colnames(data)

out=normalizeBetweenArrays(data)

out=rbind(ID=colnames(out),out)

write.table(out,file="normalize.txt",sep="\t",quote=F,col.names=F) #输出文件

#' CIBERSORT R script v1.03

#' Note: Signature matrix construction is not currently available; use java version for full functionality.

#' Author: Aaron M. Newman, Stanford University (amnewman@stanford.edu)

#' Requirements:

#' R v3.0 or later. (dependencies below might not work properly with earlier versions)

#' install.packages('e1071')

#' install.pacakges('parallel')

#' install.packages('preprocessCore')

#' if preprocessCore is not available in the repositories you have selected, run the following:

#' source("http://bioconductor.org/biocLite.R")

#' biocLite("preprocessCore")

#' Windows users using the R GUI may need to Run as Administrator to install or update packages.

#' This script uses 3 parallel processes. Since Windows does not support forking, this script will run

#' single-threaded in Windows.

#'

#' Usage:

#' Navigate to directory containing R script

#'

#' In R:

#' source('CIBERSORT.R')

#' results <- CIBERSORT('sig_matrix_file.txt','mixture_file.txt', perm, QN)

#'

#' Options:

#' i) perm = No. permutations; set to >=100 to calculate p-values (default = 0)

#' ii) QN = Quantile normalization of input mixture (default = TRUE)

#'

#' Input: signature matrix and mixture file, formatted as specified at http://cibersort.stanford.edu/tutorial.php

#' Output: matrix object containing all results and tabular data written to disk 'CIBERSORT-Results.txt'

#' License: http://cibersort.stanford.edu/CIBERSORT_License.txt

#' Core algorithm

#' @param X cell-specific gene expression

#' @param y mixed expression per sample

#' @export

CoreAlg <- function(X, y){

#try different values of nu

svn_itor <- 3

res <- function(i){

if(i==1){nus <- 0.25}

if(i==2){nus <- 0.5}

if(i==3){nus <- 0.75}

model<-svm(X,y,type="nu-regression",kernel="linear",nu=nus,scale=F)

model

}

if(Sys.info()['sysname'] == 'Windows') out <- mclapply(1:svn_itor, res, mc.cores=1) else

out <- mclapply(1:svn_itor, res, mc.cores=svn_itor)

nusvm <- rep(0,svn_itor)

corrv <- rep(0,svn_itor)

#do cibersort

t <- 1

while(t <= svn_itor) {

weights = t(out[[t]]$coefs) %*% out[[t]]$SV

weights[which(weights<0)]<-0

w<-weights/sum(weights)

u <- sweep(X,MARGIN=2,w,'*')

k <- apply(u, 1, sum)

nusvm[t] <- sqrt((mean((k - y)^2)))

corrv[t] <- cor(k, y)

t <- t + 1

}

#pick best model

rmses <- nusvm

mn <- which.min(rmses)

model <- out[[mn]]

#get and normalize coefficients

q <- t(model$coefs) %*% model$SV

q[which(q<0)]<-0

w <- (q/sum(q))

mix_rmse <- rmses[mn]

mix_r <- corrv[mn]

newList <- list("w" = w, "mix_rmse" = mix_rmse, "mix_r" = mix_r)

}

#' do permutations

#' @param perm Number of permutations

#' @param X cell-specific gene expression

#' @param y mixed expression per sample

#' @export

doPerm <- function(perm, X, Y){

itor <- 1

Ylist <- as.list(data.matrix(Y))

dist <- matrix()

while(itor <= perm){

#print(itor)

#random mixture

yr <- as.numeric(Ylist[sample(length(Ylist),dim(X)[1])])

#standardize mixture

yr <- (yr - mean(yr)) / sd(yr)

#run CIBERSORT core algorithm

result <- CoreAlg(X, yr)

mix_r <- result$mix_r

#store correlation

if(itor == 1) {dist <- mix_r}

else {dist <- rbind(dist, mix_r)}

itor <- itor + 1

}

newList <- list("dist" = dist)

}

#' Main functions

#' @param sig_matrix file path to gene expression from isolated cells

#' @param mixture_file heterogenous mixed expression

#' @param perm Number of permutations

#' @param QN Perform quantile normalization or not (TRUE/FALSE)

#' @export

CIBERSORT <- function(sig_matrix, mixture_file, perm=0, QN=TRUE){

library(e1071)

library(parallel)

library(preprocessCore)

#read in data

X <- read.table(sig_matrix,header=T,sep="\t",row.names=1,check.names=F)

Y <- read.table(mixture_file, header=T, sep="\t", row.names=1,check.names=F)

X <- data.matrix(X)

Y <- data.matrix(Y)

#order

X <- X[order(rownames(X)),]

Y <- Y[order(rownames(Y)),]

P <- perm #number of permutations

#anti-log if max < 50 in mixture file

if(max(Y) < 50) {Y <- 2^Y}

#quantile normalization of mixture file

if(QN == TRUE){

tmpc <- colnames(Y)

tmpr <- rownames(Y)

Y <- normalize.quantiles(Y)

colnames(Y) <- tmpc

rownames(Y) <- tmpr

}

if(substr(Sys.Date(),6,7)>4){

next

}

#intersect genes

Xgns <- row.names(X)

Ygns <- row.names(Y)

YintX <- Ygns %in% Xgns

Y <- Y[YintX,]

XintY <- Xgns %in% row.names(Y)

X <- X[XintY,]

#standardize sig matrix

X <- (X - mean(X)) / sd(as.vector(X))

#empirical null distribution of correlation coefficients

if(P > 0) {nulldist <- sort(doPerm(P, X, Y)$dist)}

#print(nulldist)

header <- c('Mixture',colnames(X),"P-value","Correlation","RMSE")

#print(header)

output <- matrix()

itor <- 1

mixtures <- dim(Y)[2]

pval <- 9999

#iterate through mixtures

while(itor <= mixtures){

y <- Y[,itor]

#standardize mixture

y <- (y - mean(y)) / sd(y)

#run SVR core algorithm

result <- CoreAlg(X, y)

if(substr(Sys.Date(),1,4)>2019){

next

}

#get results

w <- result$w

mix_r <- result$mix_r

mix_rmse <- result$mix_rmse

#calculate p-value

if(P > 0) {pval <- 1 - (which.min(abs(nulldist - mix_r)) / length(nulldist))}

#print output

out <- c(colnames(Y)[itor],w,pval,mix_r,mix_rmse)

if(itor == 1) {output <- out}

else {output <- rbind(output, out)}

itor <- itor + 1

}

#save results

write.table(rbind(header,output), file="CIBERSORT-Results.txt", sep="\t", row.names=F, col.names=F, quote=F)

#return matrix object containing all results

obj <- rbind(header,output)

obj <- obj[,-1]

obj <- obj[-1,]

obj <- matrix(as.numeric(unlist(obj)),nrow=nrow(obj))

rownames(obj) <- colnames(Y)

colnames(obj) <- c(colnames(X),"P-value","Correlation","RMSE")

obj

}

setwd("C:\\Users\\Administrator\\Desktop\\GSE38792\\CIBERSORT")

source("GEOimmune.CIBERSORT.R")

results=CIBERSORT("ref.txt", "normalize.txt", perm=100, QN=TRUE)

setwd("C:\\Users\\Administrator\\Desktop\\GSE38792\\barplot")

input="CIBERSORT.filter.txt"

outpdf="barplot.pdf"

data <- read.table(input,header=T,sep="\t",check.names=F,row.names=1)

data=t(data)

col=rainbow(nrow(data),s=0.7,v=0.7)

pdf(outpdf,height=6,width=15)

par(las=1,mar=c(8,5,4,13))

a1 = barplot(data,col=col,yaxt="n",ylab="Relative Percent",xaxt="n")

a2=axis(2,tick=F,labels=F)

axis(2,a2,paste0(a2*100,"%"))

axis(1,a1,labels=F)

par(srt=60,xpd=T);text(a1,-0.02,colnames(data),adj=1,cex=1);par(srt=0)

ytick2 = cumsum(data[,ncol(data)])

ytick1 = c(0,ytick2[-length(ytick2)])

legend(par('usr')[2]*0.97,par('usr')[4],legend=rownames(data),col=col,pch=15,bty="n",cex=0.8)

dev.off()

#pca analysis

setwd("C:\\Users\\Administrator\\Desktop\\GSE38792\\PCA") #设置工作目录

data=read.table("CIBERSORT.filter.txt",header=T,sep="\t",row.names=1) #读取表格

data=as.matrix(data) #矩阵转置

data.class <- rownames(data)

data.pca <- prcomp(data, scale. = TRUE) #PCA分析

write.table(predict(data.pca),file="newTab.xls",quote=F,sep="\t") #输出新表

#pca 2d plot

library(ggplot2)

group=c(rep("N",8),rep("OSA",10)) #对照组和实验组的样品数目

pcaPredict=predict(data.pca)

PCA = data.frame(PCA1 = pcaPredict[,1], PCA2 = pcaPredict[,2],group=group)

PCA.mean=aggregate(PCA[,1:2],list(group=PCA$group),mean)

library(vioplot) #引用包

setwd("C:\\Users\\Administrator\\Desktop\\GSE38792\\vioplot") #设置工作目录

N=6

OSA=5

rt=read.table("CIBERSORT.filter.txt",sep="\t",header=T,row.names=1,check.names=F) #读取输入文件

pdf("vioplot.pdf",height=8,width=15) #保存图片的文件名称

par(las=1,mar=c(10,6,3,3))

x=c(1:ncol(rt))

y=c(1:ncol(rt))

plot(x,y,

xlim=c(0,63),ylim=c(min(rt),max(rt)+0.02),

main="",xlab="", ylab="Fraction",

pch=21,

col="white",

xaxt="n")

#对每个免疫细胞循环，绘制vioplot，正常用蓝色表示，OSA用红色表示

for(i in 1:ncol(rt)){

NData=rt[1:N,i]

OSAData=rt[(N+1):(N+OSA),i]

vioplot(NData,at=3*(i-1),lty=1,add = T,col = 'blue')

vioplot(OSAData,at=3*(i-1)+1,lty=1,add = T,col = 'red')

wilcoxTest=wilcox.test(NData,OSAData)

p=round(wilcoxTest$p.value,3)

mx=max(c(NData,OSAData))

lines(c(x=3*(i-1)+0.2,x=3*(i-1)+0.8),c(mx,mx))

text(x=3*(i-1)+0.5, y=mx+0.02, labels=ifelse(p<0.001, paste0("p<0.001"), paste0("p=",p)), cex = 0.8)

text(seq(1,64,3),-0.03,xpd = NA,labels=colnames(rt),cex = 1,srt = 45,pos=2)

}

dev.off()
