# Supplementary material for: CD40LG and GZMB were correlated with adipose tissue macrophage infiltration and involved in obstructive sleep apnea related metabolic dysregulation: Evidence from bioinformatics analysis
Source: Front Genet. 2023 Feb 27;14:1128139. doi: 10.3389/fgene.2023.1128139 (PMC10009156; doi:10.3389/fgene.2023.1128139)
Supplement: Supplementary file 3 [file Table3.DOCX]

Supplementary Material

# Supplementary Figures and Tables

## Supplementary Figure


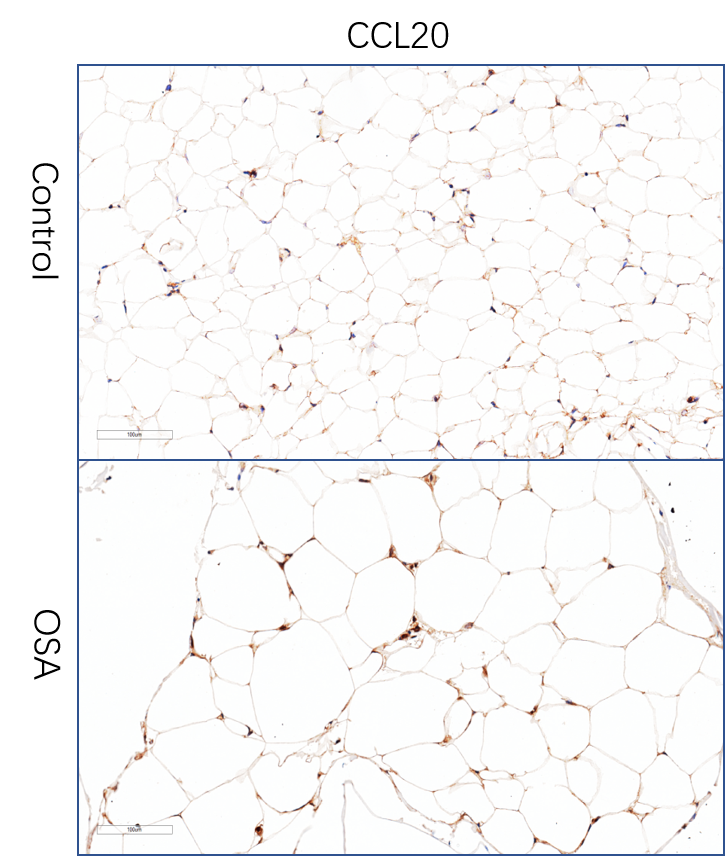


**Supplementary Figure S1.** Representative immunohistochemistry images of CCL20 in VAT between OSA and control patients. OSA, obstructive sleep apnea; VAT, visceral adipose tissue.


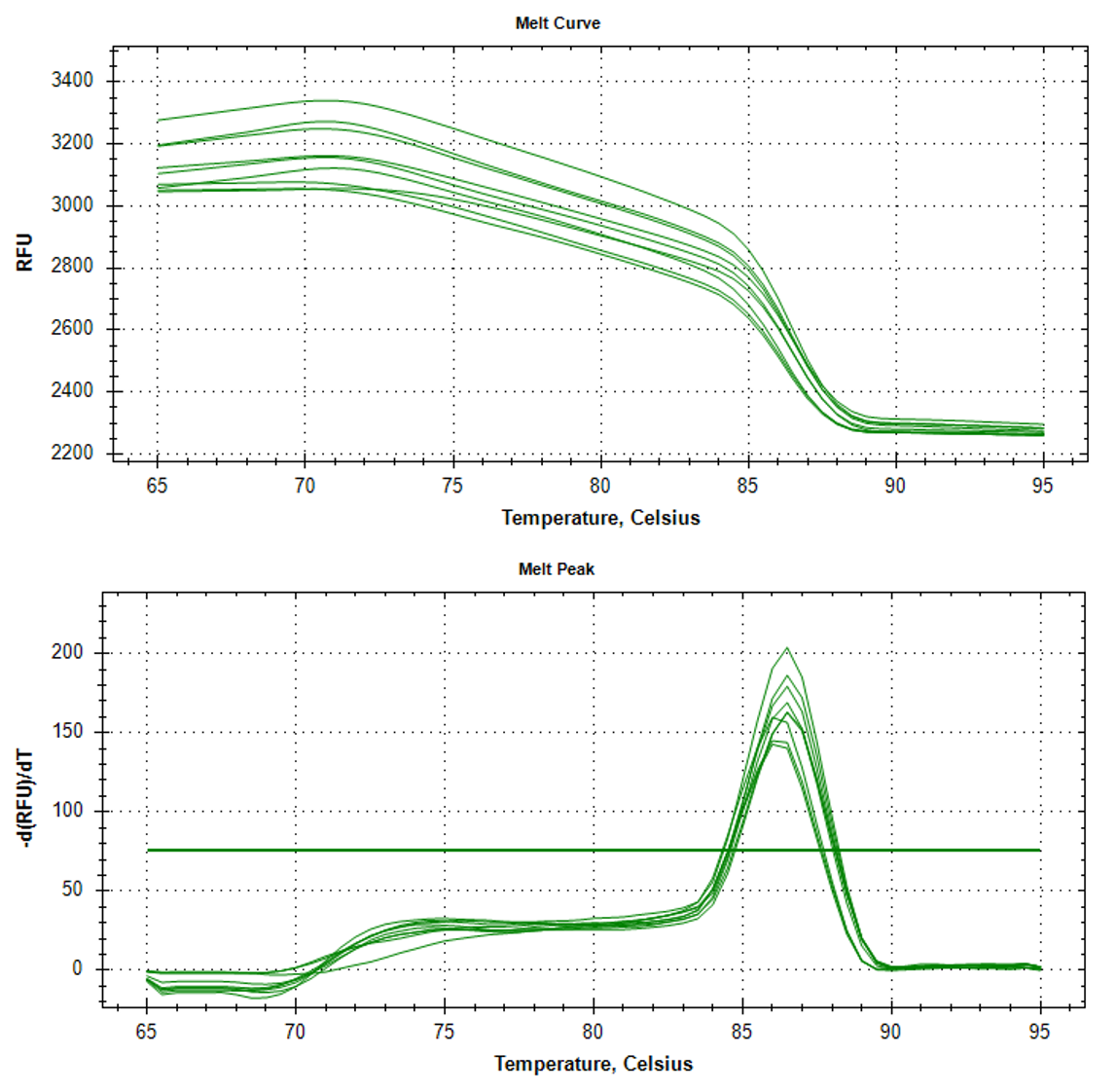


**Supplementary Figure S2.** Representative graph of melting temperature of PCR results.


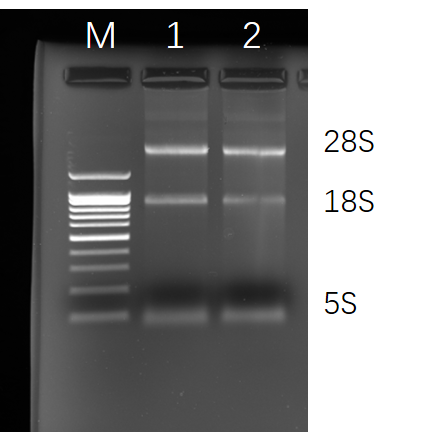


**Supplementary Figure S3.** Representative graph of electrophoretic results for examining RNA integrity.

## Supplementary Table

| **Supplementary table S1**. Sequences of hub genes and markers of macrophages. | | |
| --- | --- | --- |
|  | | |
| **Hub genes** | | **Primer Sequences (5'→3‘)** |
| *IL1R1* (human) | Forward | CGTCCCTGTCCTCTTAACCCAAATG |
|  | Reverse | GTAATGTCCTGAATCCTCCACCTTAGC |
| *CRP* (human) | Forward | GCCACCAAGAGACAAGACAATGAGA |
|  | Reverse | AATGTGTACTGGAGCTACTGTGACTTC |
| *IL33* (human) | Forward | CAGGTGACGGTGTTGATGGTAAGAT |
|  | Reverse | GCTCCACAGAGTGTTCCTTGTTGT |
| *CD40LG* (human) | Forward | AGGATACTACACCATGAGCAACAACT |
|  | Reverse | CCGATTGGAACAGAAGGTGACTTG |
| *CCL5* (human) | Forward | CAGCAGTCGTCCACAGGTCAAG |
|  | Reverse | ACTCTCCATCCTAGCTCATCTCCAA |
| *CCL20* (human) | Forward | TACTCCACCTCTGCGGCGAAT |
|  | Reverse | GATGAAGAATACGGTCTGTGTATCCAAG |
| *CXCL8* (human) | Forward | TCTCTTGGCAGCCTTCCTGAT |
|  | Reverse | TGGTCCACTCTCAATCACTCTCA |
| *TLR3* (human) | Forward | TCAACGACTGATGCTCCGAAGG |
|  | Reverse | AAGTTACGAAGAGGCTGGAATGGT |
| *TSLP* (human) | Forward | CGAGTTCAACAACACCGTCTCTTG |
|  | Reverse | TCCTCTTCTTCATTGCCTGAGTAGC |
| *GZMB* (human) | Forward | TGCGGTGGCTTCCTGATACG |
|  | Reverse | GAAGTTCTTAGGATTATAGGCTGGATGG |
| *ACTB* (human) | Forward | GGCACCACACCTTCTACAATGAG |
|  | Reverse | GGATAGCACAGCCTGGATAGCA |
| **M1 marker** |  |  |
| *CD11C* (human) | Forward | CGACCATATCTGCCAGGACAATCTC |
|  | Reverse | CCACACCATCACTTCTGCGTTCA |
| **M2 marker** |  |  |
| *CD206* (human) | Forward | GCTGACTGTGTTGTTATTATTGGAGGT |
|  | Reverse | GGTCGGATCGTGTCTGGCATAT |

Abbreviations: *IL1R1*, Interleukin 1 Receptor Type 1; *CRP*, C-Reactive Protein; *IL33*, Interleukin 33; *CD40LG*, CD40 Ligand; *CCL5*, C-C Motif Chemokine Ligand 5; *CCL20*, C-C Motif Chemokine Ligand 20; *CXCL8*, C-X-C Motif Chemokine Ligand 8; *TLR3*, Toll Like Receptor 3; *TSLP*, Thymic Stromal Lymphopoietin; *GZMB*, Granzyme B; ACTB, Actin Beta; *CD11c*, Integrin Subunit Alpha X; *CD206*, Mannose Receptor C-Type 1.

**Supplementary table S2.** The functional annotation of 10 hub genes.

| **No** | **Gene** | **Full name** | **Function** |
| --- | --- | --- | --- |
| 1 | *IL1R1* | Interleukin 1 Receptor Type 1 | Receptor for IL1A, IL1B and IL1RN. It is an important mediator involved in many cytokine-induced immune and inflammatory responses |
| 2 | *CRP* | C-Reactive Protein | It promotes agglutination, bacterial capsular swelling, phagocytosis and complement fixation through its calcium-dependent binding to phosphorylcholine. |
| 3 | *IL33* | Interleukin 33 | A cytokine that binds to the IL1RL1/ST2 receptor. It is involved in the maturation of Th2 cells and the activation of mast cells, basophils, eosinophils and natural killer cells. |
| 4 | *CD40LG* | CD40 Ligand | It is expressed on the surface of T cells. It regulates B cell function by engaging CD40 on the B cell surface. |
| 5 | *CCL5* | C-C Motif Chemokine Ligand 5 | One chemokine belongs to the CC subfamily, functions as a chemoattractant for blood monocytes, memory T helper cells and eosinophils. It is involved in immunoregulatory and inflammatory. |
| 6 | *CCL20* | C-C Motif Chemokine Ligand 20 | Acts as a ligand for C-C chemokine receptor CCR6. It belongs to the subfamily of small cytokine and involved in immunoregulatory and inflammatory. |
| 7 | *CXCL8* | C-X-C Motif Chemokine Ligand 8 | It is a member of the CXC chemokine family and is a major mediator of the inflammatory response. It functions as a chemotactic factor by guiding the neutrophils to the site of infection. |
| 8 | *TLR3* | Toll Like Receptor 3 | It is a member of the Toll-like receptor (TLR) family which plays a fundamental role in pathogen recognition and activation of innate immunity. |
| 9 | *TSLP* | Thymic Stromal Lymphopoietin | One hemopoietic cytokine mainly impacts myeloid cells and induces the release of T cell-attracting chemokines from monocytes and enhances the maturation of CD11c(+) dendritic cells. |
| 10 | *GZMB* | Granzyme B | It is secreted by natural killer (NK) cells and cytotoxic T lymphocytes (CTLs) and proteolytically processed to generate the active protease, which induces target cell apoptosis. |

Abbreviations: No, number; IL1A, Interleukin 1 Alpha; IL1B, Interleukin 1 Beta; IL1RN, Interleukin 1 Receptor Antagonist; IL1RL1, Interleukin 1 Receptor Like 1; CD40, CD40 Molecule; CCR6, C-C Motif Chemokine Receptor 6.

**Supplementary table S3.** GO analyses results of immune-related Hub genes (Top 5 of each category according to P value). "Count" means how many DEGs were involved.

| **Category** | **ID** | **Description** | **GeneRatio** | **pvalue** | **Gene symbol** | **Count** |
| --- | --- | --- | --- | --- | --- | --- |
| MF | GO:0005125 | cytokine activity | 0.6 | 8.3226E-10 | IL33/CD40LG/CXCL8/CCL5/TSLP/CCL20 | 6 |
| MF | GO:0005126 | cytokine receptor binding | 0.6 | 1.9609E-09 | IL33/CD40LG/CXCL8/CCL5/TSLP/CCL20 | 6 |
| MF | GO:0048018 | receptor ligand activity | 0.6 | 6.4977E-08 | IL33/CD40LG/CXCL8/CCL5/TSLP/CCL20 | 6 |
| MF | GO:0030546 | signaling receptor activator activity | 0.6 | 6.904E-08 | IL33/CD40LG/CXCL8/CCL5/TSLP/CCL20 | 6 |
| MF | GO:0008009 | chemokine activity | 0.3 | 2.1184E-06 | CXCL8/CCL5/CCL20 | 3 |
| CC | GO:0036020 | endolysosome membrane | 0.1 | 0.00713646 | TLR3 | 1 |
| CC | GO:0036019 | endolysosome | 0.1 | 0.01018088 | TLR3 | 1 |
| CC | GO:0005720 | nuclear heterochromatin | 0.1 | 0.01725189 | IL33 | 1 |
| CC | GO:0009897 | external side of plasma membrane | 0.2 | 0.01822122 | IL1R1/CD40LG | 2 |
| CC | GO:0001772 | immunological synapse | 0.1 | 0.01976621 | GZMB | 1 |
| BP | GO:0002685 | regulation of leukocyte migration | 0.5 | 4.1147E-08 | IL1R1/IL33/CXCL8/CCL5/CCL20 | 5 |
| BP | GO:1990266 | neutrophil migration | 0.4 | 3.3919E-07 | IL1R1/CXCL8/CCL5/CCL20 | 4 |
| BP | GO:0002687 | positive regulation of leukocyte migration | 0.4 | 6.2373E-07 | IL1R1/CXCL8/CCL5/CCL20 | 4 |
| BP | GO:0002700 | regulation of production of molecular mediator of immune response | 0.4 | 6.5968E-07 | IL1R1/IL33/CD40LG/TLR3 | 4 |
| BP | GO:0097530 | granulocyte migration | 0.4 | 7.7681E-07 | IL1R1/CXCL8/CCL5/CCL20 | 4 |

Abbreviations: GO, Gene Ontology; DEGs, Differentially Expressed Genes; MF, Molecular Function; CC, Cell Component; BP, Biological Process; *IL33*, Interleukin 33;*CD40LG*, CD40 Ligand; *CXCL8*, C-X-C Motif Chemokine Ligand 8;*CCL5*, C-C Motif Chemokine Ligand 5; *TSLP*, Thymic Stromal Lymphopoietin; *CCL20*, C-C Motif Chemokine Ligand 20; *TLR3*, Toll Like Receptor 3; *IL33*, Interleukin 33; *IL1R1*, Interleukin 1 Receptor Type 1; *GZMB*, *GZMB*, Granzyme B.

**Supplementary table S4**. Kyoto Encyclopedia of Genes and Genomes (KEGG) pathway analysis of immune-related hub genes. (Top 14 according to P value). "Count" means how many DEGs were involved.

| **ID** | **Description** | **GeneRatio** | **pvalue** | **Gene symbol** | **Count** |
| --- | --- | --- | --- | --- | --- |
| hsa04060 | Asthma | 0.11 | 0.04638335 | CD40LG | 1 |
| hsa05323 | Allograft rejection | 0.22 | 0.001491646 | CD40LG/GZMB | 2 |
| hsa04620 | Malaria | 0.22 | 0.00254228 | CD40LG/CXCL8 | 2 |
| hsa05330 | Autoimmune thyroid disease | 0.22 | 0.002846552 | CD40LG/GZMB | 2 |
| hsa04062 | Cytosolic DNA-sensing pathway | 0.22 | 0.003058518 | IL33/CCL5 | 2 |
| hsa05144 | NOD-like receptor signaling pathway | 0.22 | 0.003390053 | CXCL8/CCL5 | 2 |
| hsa05320 | Epithelial cell signaling in Helicobacter pylori infection | 0.22 | 0.004481342 | CXCL8/CCL5 | 2 |
| hsa04623 | Chagas disease (American trypanosomiasis) | 0.22 | 0.010239519 | CXCL8/CCL5 | 2 |
| hsa04621 | Amoebiasis | 0.22 | 0.010622191 | IL1R1/CXCL8 | 2 |
| hsa05120 | Hepatitis C | 0.22 | 0.01687128 | CXCL8/TLR3 | 2 |
| hsa05142 | Rheumatoid arthritis | 0.33 | 0.000288865 | CXCL8/CCL5/CCL20 | 3 |
| hsa05146 | Toll-like receptor signaling pathway | 0.33 | 0.000391922 | CXCL8/CCL5/TLR3 | 3 |
| hsa05160 | Chemokine signaling pathway | 0.33 | 0.002363307 | CXCL8/CCL5/CCL20 | 3 |
| hsa05310 | Cytokine-cytokine receptor interaction | 0.67 | 5.85606E-07 | IL1R1/CD40LG/CXCL8/CCL5/TSLP/CCL20 | 6 |

Abbreviations: KEGG, Kyoto Encyclopedia of Genes and Genomes; *IL33*, Interleukin 33;*CD40LG*, CD40 Ligand; *CXCL8*, C-X-C Motif Chemokine Ligand 8;*CCL5*, C-C Motif Chemokine Ligand 5; *TSLP*, Thymic Stromal Lymphopoietin; *CCL20*, C-C Motif Chemokine Ligand 20; *TLR3*, Toll Like Receptor 3; *IL33*, Interleukin 33; *IL1R1*, Interleukin 1 Receptor Type 1; *GZMB*, *GZMB*, Granzyme B.

**Supplementary table S5.** The transcription factors (TFs) regulated network in OSA patients.

| **TF** | **NES** | **mRNA** | **Correlation coefficient (r)** | **p value** | **Immune cells** |
| --- | --- | --- | --- | --- | --- |
| NR2F2 | 7.525 | CCL20 | 0.772 | 0.005 | Monocytes |
| NR2F2 | 7.525 | CCL20 | -0.722 | 0.012 | Macrophages M1 |
| FOXA1 | 6.746 | CCL20 | 0.772 | 0.005 | Monocytes |
| FOXA1 | 6.746 | CCL20 | -0.722 | 0.012 | Macrophages M1 |
| HDAC2 | 6.388 | CCL20 | 0.772 | 0.005 | Monocytes |
| HDAC2 | 6.388 | CCL20 | -0.722 | 0.012 | Macrophages M1 |
| EP300 | 6.207 | CCL20 | 0.772 | 0.005 | Monocytes |
| EP300 | 6.207 | CCL20 | -0.722 | 0.012 | Macrophages M1 |
| TEAD4 | 6.207 | CCL20 | 0.772 | 0.005 | Monocytes |
| TEAD4 | 6.207 | CCL20 | -0.722 | 0.012 | Macrophages M1 |
| CEBPB | 5.486 | CCL20 | 0.772 | 0.005 | Monocytes |
| CEBPB | 5.486 | CCL20 | -0.722 | 0.012 | Macrophages M1 |
| FOXA2 | 5.193 | CCL20 | 0.772 | 0.005 | Monocytes |
| FOXA2 | 5.193 | CCL20 | -0.722 | 0.012 | Macrophages M1 |
| RXRA | 5.086 | CCL20 | 0.772 | 0.005 | Monocytes |
| RXRA | 5.086 | CCL20 | -0.722 | 0.012 | Macrophages M1 |
| NFIC | 5.017 | CCL20 | 0.772 | 0.005 | Monocytes |
| NFIC | 5.017 | CCL20 | -0.722 | 0.012 | Macrophages M1 |
| NR2F2 | 7.525 | CCL5 | 0.665 | 0.026 | Macrophages M1 |
| GATA2 | 5.428 | CCL5 | 0.665 | 0.026 | Macrophages M1 |
| RCOR1 | 5.278 | CCL5 | 0.665 | 0.026 | Macrophages M1 |
| NR2F2 | 7.525 | CD40LG | 0.787 | 0.004 | T cells CD8 |
| NR2F2 | 7.525 | CD40LG | -0.694 | 0.018 | T cells CD4 |
| NR2F2 | 7.525 | CD40LG | -0.720 | 0.013 | Macrophages M0 |
| NR2F2 | 7.525 | CRP | 0.713 | 0.014 | Neutrophils |
| NR2F2 | 7.525 | CRP | -0.660 | 0.027 | Mast cells resting |
| NR2F2 | 7.525 | CRP | -0.680 | 0.021 | Macrophages M2 |
| NR2F2 | 7.525 | IL1R1 | -0.622 | 0.041 | Macrophages M0 |
| NR2F2 | 7.525 | IL1R1 | -0.645 | 0.032 | T cells CD4 |
| NR2F2 | 7.525 | IL1R1 | -0.779 | 0.005 | Neutrophils |
| FOXA1 | 6.746 | IL1R1 | -0.622 | 0.041 | Macrophages M0 |
| FOXA1 | 6.746 | IL1R1 | -0.645 | 0.032 | T cells CD4 |
| FOXA1 | 6.746 | IL1R1 | -0.779 | 0.005 | Neutrophils |
| NFIC | 5.017 | IL1R1 | -0.622 | 0.041 | Macrophages M0 |
| NFIC | 5.017 | IL1R1 | -0.645 | 0.032 | T cells CD4 |
| NFIC | 5.017 | IL1R1 | -0.779 | 0.005 | Neutrophils |
| HDAC2 | 6.388 | IL1R1 | -0.622 | 0.041 | Macrophages M0 |
| HDAC2 | 6.388 | IL1R1 | -0.645 | 0.032 | T cells CD4 |
| HDAC2 | 6.388 | IL1R1 | -0.779 | 0.005 | Neutrophils |
| EP300 | 6.207 | IL1R1 | -0.622 | 0.041 | Macrophages M0 |
| EP300 | 6.207 | IL1R1 | -0.645 | 0.032 | T cells CD4 |
| EP300 | 6.207 | IL1R1 | -0.779 | 0.005 | Neutrophils |
| TEAD4 | 6.207 | IL1R1 | -0.622 | 0.041 | Macrophages M0 |
| TEAD4 | 6.207 | IL1R1 | -0.645 | 0.032 | T cells CD4 |
| TEAD4 | 6.207 | IL1R1 | -0.779 | 0.005 | Neutrophils |
| CEBPB | 5.486 | IL1R1 | -0.622 | 0.041 | Macrophages M0 |
| CEBPB | 5.486 | IL1R1 | -0.645 | 0.032 | T cells CD4 |
| CEBPB | 5.486 | IL1R1 | -0.779 | 0.005 | Neutrophils |
| FOXA2 | 5.193 | IL1R1 | -0.622 | 0.041 | Macrophages M0 |
| FOXA2 | 5.193 | IL1R1 | -0.645 | 0.032 | T cells CD4 |
| FOXA2 | 5.193 | IL1R1 | -0.779 | 0.005 | Neutrophils |
| RXRA | 5.086 | IL1R1 | -0.622 | 0.041 | Macrophages M0 |
| RXRA | 5.086 | IL1R1 | -0.645 | 0.032 | T cells CD4 |
| RXRA | 5.086 | IL1R1 | -0.779 | 0.005 | Neutrophils |
| GATA2 | 5.428 | IL33 | 0.689 | 0.019 | Macrophages M2 |
| GATA2 | 5.428 | IL33 | -0.603 | 0.049 | T cells CD4 |
| GATA2 | 5.428 | IL33 | -0.625 | 0.040 | Macrophages M0 |
| GATA2 | 5.428 | IL33 | -0.816 | 0.002 | Neutrophils |
| RCOR1 | 5.278 | IL33 | 0.689 | 0.019 | Macrophages M2 |
| RCOR1 | 5.278 | IL33 | -0.603 | 0.049 | T cells CD4 |
| RCOR1 | 5.278 | IL33 | -0.625 | 0.040 | Macrophages M0 |
| RCOR1 | 5.278 | IL33 | -0.816 | 0.002 | Neutrophils |
| NR2F2 | 7.525 | TSLP | 0.748 | 0.008 | T cells CD4 |
| NR2F2 | 7.525 | TSLP | 0.708 | 0.015 | Neutrophils |
| NR2F2 | 7.525 | TSLP | -0.632 | 0.037 | T cells CD8 |
| GATA2 | 5.428 | TSLP | 0.748 | 0.008 | T cells CD4 |
| GATA2 | 5.428 | TSLP | 0.708 | 0.015 | Neutrophils |
| GATA2 | 5.428 | TSLP | -0.632 | 0.037 | T cells CD8 |

Abbreviations: NR2F2, Nuclear Receptor Subfamily 2 Group F Member 2; FOXA1, Forkhead Box A1 ; HDAC2, Histone Deacetylase 2 ;EP300, E1A Binding Protein P300; TEAD4, TEA Domain Transcription Factor 4 ;CEBPB, CCAAT Enhancer Binding Protein Beta ;FOXA2, Forkhead Box A2; RXRA, Retinoid X Receptor Alpha ; NFIC, Nuclear Factor I C; RCOR1, REST Corepressor 1; GATA2, GATA Binding Protein 2.
